# W_2_C/WS_2_ Alloy Nanoflowers as Anode Materials for Lithium-Ion Storage

**DOI:** 10.3390/nano10071336

**Published:** 2020-07-09

**Authors:** Thang Phan Nguyen, Il Tae Kim

**Affiliations:** Department of Chemical and Biological Engineering, Gachon University, Seongnam-si, Gyeonggi-do 13120, Korea; phanthang87@gmail.com

**Keywords:** W_2_C, WS_2_, hydrothermal, nanoflowers, lithium-ion batteries

## Abstract

Recently, composites of MXenes and two-dimensional transition metal dichalcogenides have emerged as promising materials for energy storage applications. In this study, W_2_C/WS_2_ alloy nanoflowers (NFs) were prepared by a facile hydrothermal method. The alloy NFs showed a particle size of 200 nm–1 μm, which could be controlled. The electrochemical performance of the as-prepared alloy NFs was investigated to evaluate their potential for application as lithium-ion battery (LIB) anodes. The incorporation of W_2_C in the WS_2_ NFs improved their electronic properties. Among them, the W_2_C/WS_2__4h NF electrode showed the best electrochemical performance with an initial discharge capacity of 1040 mAh g^−1^ and excellent cyclability corresponding to a reversible capacity of 500 mAh g^−1^ after 100 cycles compared to that of the pure WS_2_ NF electrode. Therefore, the incorporation of W_2_C is a promising approach to improve the performance of LIB anode materials.

## 1. Introduction

Two-dimensional (2D) materials such as graphene and transition metal chalcogenides show great potential for energy storage and conversion applications owing to their large surface area, high conductivity, and good physical and chemical stability [1,2,3,4,5,6,7,8,9,10,11,12]. Among these 2D materials, MXenes, which have been discovered recently, have been extensively investigated for energy storage applications. It should be noted that MXenes are the transition metal carbides/nitride, which have graphene-like structure, possessing many advantages of 2D materials such as high conductivity, flexibility, easy processing, and so on [13]. Mashtalir et al. synthesized intercalated Ti_3_C_2_ flakes with high stability and the charging rate for application as an anode material for lithium-ion batteries (LIBs) [14]. Naguib et al. prepared niobium and vanadium carbide LIB anodes with high rate capacity [15]. The MXene anodes exhibited excellent electrochemical performance at high currents because of their low diffusion barrier [16]. In addition, Fe_3_O_4_@Ti_2_C_3_ LIB anodes exhibited an ultrahigh capacity of approximately 747 mAh g^−1^ at 1 C for 1000 cycles. Moreover, these materials exhibited a capacity of approximately 278 mAh g^−1^ at the high rate of 5 C [17]. Zhang et al. have demonstrated the use of MXenes as conductive binders for viscous aqueous inks of silicon materials, which were used as high-capacity anode materials for LIBs [18]. In these materials, MXenes not only acted as a conductive network for Si particles, but also improved the mechanical stability of the material.

Tungsten metal compounds such as oxides, chalcogenides, and carbides are used in a wide range of applications such as catalysis, energy conversion, and energy storage [19,20,21,22,23,24,25,26,27]. Feng et al. prepared WS_2_ nanoflakes with a high reversible capacity of 680 mAh g^−1^ for 20 cycles as anode materials for LIBs [27]. Srinivaas et al. have prepared highly rich 1T WS_2_ phase in few layered nanoflowers (NFs) with stable electrochemical performance and a high initial capacity of approximately 890 mAh g^−1^ as anode materials for LIBs [28]. W_2_C is a good catalyst. When grown on carbon nanotubes, it exhibits high photo/electrocatalytic performance for the hydrogen evolution reaction [26,29]. Simulation results have shown that W_2_C can exhibit an ultra-fast loading of lithium ions owing to its low diffusion barrier of about 0.045–0.13 eV [30]. Thus, the combination of WS_2_ and W_2_C is expected to yield a promising LIB anode material.

In this study, we prepared a W_2_C/WS_2_ NF composite via a facile hydrothermal process. By controlling the reaction time, alloy NFs with different sizes could be prepared. The structures, chemical compositions, and binding states of the as-prepared NFs were investigated. Finally, the potential of the W_2_C/WS_2_ NFs for LIB anode applications was investigated. The NFs showed promising electrochemical performance and excellent Li storage.

## 2. Materials and Methods 

### 2.1. Chemical Materials

Tungsten (VI) chloride (WCl_6_, 99.9% trace metals basis, Sigma-Aldrich Inc., St. Louis, MO, USA), thioacetamide (C_2_H_5_NS, 99%, Sigma-Aldrich, Sigma-Aldrich Inc., St. Louis, MO, USA), amorphous carbon black Super-P (C, approximately 40 nm 99.99%, Alfa Aesar Inc., MA, USA), absolute ethanol (C_2_H_5_OH, Alpha Aesar Inc., MA, USA), and polyvinylidene fluoride (PVDF, 534,000 M_W_, Sigma-Aldrich, Sigma-Aldrich Inc., St. Louis, MO, USA) were used as received without any treatment.

### 2.2. Synthesis of W_2_C/WS_2_ NFs

The W_2_C/WS_2_ alloy NFs were prepared according to a previously reported procedure [31]. Briefly, 0.6 g of WCl_6_ was dispersed as the W source in a 20 mL tube with 4 mL of absolute ethanol. For the sulfur and carbon source, 1.2 g of thioacetamide was dispersed in 4 mL of absolute ethanol under stirring. Then, the WCl_6_ solution was quickly added to the thioacetamide solution, and the resulting solution was stirred for 5 min. Then, 30 mL of deionized (DI) water was added, and the solution was stirred for 1 h. Then, the reaction mixture was transferred to a 50 mL polypropylene-lined autoclave and heated in an oven at 250 °C for 2, 4, and 12 h to obtain the W_2_C/WS_2__2h, W_2_C/WS_2__4h, and W_2_C/WS_2__12h precipitates, respectively. These precipitates were washed four times with ethanol and DI water and were then dried in an oven at 60 °C to obtain the W_2_C/WS_2_ powders.

### 2.3. Characterization

X-ray diffraction (XRD) (D/MAX-2200 Rigaku, Tokyo, Japan) was used to analyze the structure of the samples. The XRD patterns of the samples were recorded over the 2θ range of 5–70°. The morphologies, structures, and sizes of the samples were investigated by scanning electron microscopy (SEM) (Hitachi S4700, Tokyo, Japan) and transmission electron microscopy (TEM) (TECNAI G2F30, FEI corp., OR, USA). The Raman spectra of the samples were acquired using a Raman spectrometer (Lab RAM HR, Horiba JobinYvon, Horiba Ltd., Kyoto, Japan, 532 nm laser excitation). X-ray photoelectron spectroscopy (XPS) (Axis Ultra DLD, Kratos Analytical Ltd, Kyoto, Japan) under a high vacuum of 1.6 × 10^−10^ mbar with a monochromatic Al Kα line was used to investigate the chemical compositions and atomic binding of the samples. 

### 2.4. Electrochemical Measurements

The LIBs were assembled using coin-type cells (CR 2032, Rotech Inc., Gwangju, Korea). The working electrode was prepared by casting a slurry of 70% active material (W_2_C/WS_2_ alloy NFs), 15% conductive carbon black (Super-P), and 15% PVDF in N-methyl-2-pyrrolidinone on a copper foil by doctor blading. After drying in a vacuum oven at 70 °C for 12 h, the electrodes were punched into circular discs with a diameter of 12 mm. The battery half-cell structures were assembled under an Ar_2_ atmosphere in a glovebox. A lithium foil, polyethylene, and 1M LiPF_6_ in ethylene carbonate/diethylene carbonate (1:1 in volume) were employed as the reference electrode, separator, and electrolyte, respectively. Galvanostatic electrochemical charge–discharge measurements were carried out using a battery cycle tester (WBCS3000, WonAtech, Seocho-gu, Seoul) over the voltage range of 0.1–3.0 V versus Li/Li^+^. Cyclic voltammetry (CV) tests were carried out using ZIVE MP1 (WonAtech, Seocho-gu, Seoul) over the voltage range of 0.1–3.0 V at a scanning rate of 0.1 mV s^−1^. Electrochemical impedance spectroscopic (EIS) measurements were carried out using a ZIVE MP1 (WonAtech, Seocho-gu, Seoul) over the frequency range of 100 kHz–0.1 Hz.

## 3. Results

**Figure 1:**
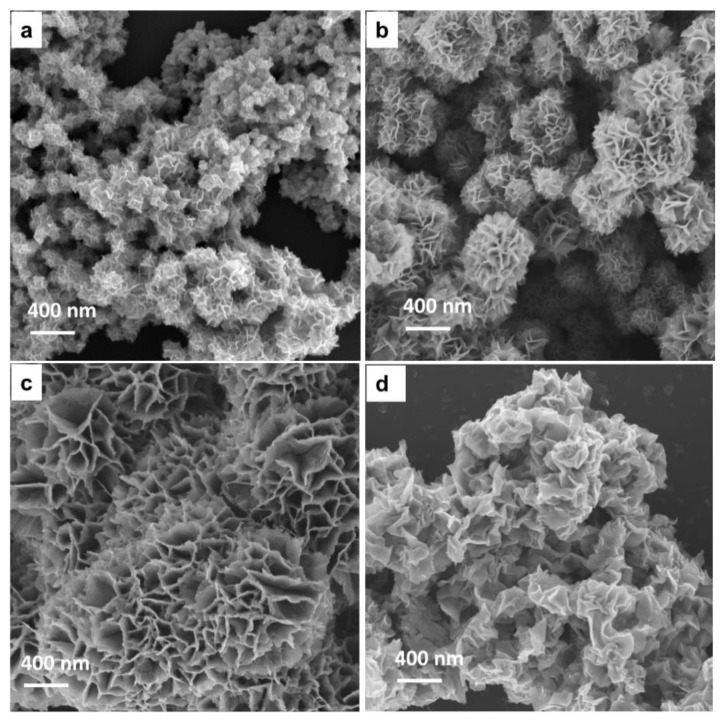
SEM images of the (**a**) W_2_C/WS_2__2h, (**b**) W_2_C/WS_2__4h, (**c**) W_2_C/WS_2__12h, and (**d**) WS_2_ NFs.

For the synthesis of the W_2_C/WS_2_ NFs, the WCl_6_ precursor was pre-mixed with absolute ethanol (WCl_6_ + xC_2_H_5_OH → WCl_6-x_(OC_2_H_5_)_x_ + xHCl) before mixing with thioacetamide to prevent its unexpected reaction with moisture (WCl_6_ + xH_2_O → WCl_6-x_(OH)_x_ + xHCl) [31,32]. The hydrolysis of thioacetamide produced hydrosulfide, acetic acid, and ammonia (2C_3_H_5_NS + 6H_2_O → 2H_2_S + 3CH_3_COOH + 2NH_3_). The introduction of hydrosulfide, acetic acid, and ethanol, respectively, acted as the sulfur and carbon sources for the formation of the W_2_C/WS_2_ alloys. Figure 1a–c show the SEM images of the W_2_C/WS_2__2h, W_2_C/WS_2__4h, and W_2_C/WS_2__12h NFs, respectively. The particle sizes of the W_2_C/WS_2__2h, W_2_C/WS_2__4h, and W_2_C/WS_2__12h NFs were 200, 400, and 1000 nm, respectively. The NFs showed a highly uniform structure, indicating that the synthesis procedure was highly reproducible. Each NF consisted of several leaves of W_2_C nanosheets and WS_2_ nanocrystals. Meanwhile, the WS_2_ NFs synthesized without ethanol showed a complex structure because of the non-uniform dispersion of W (Figure 1d).

**Figure 2:**
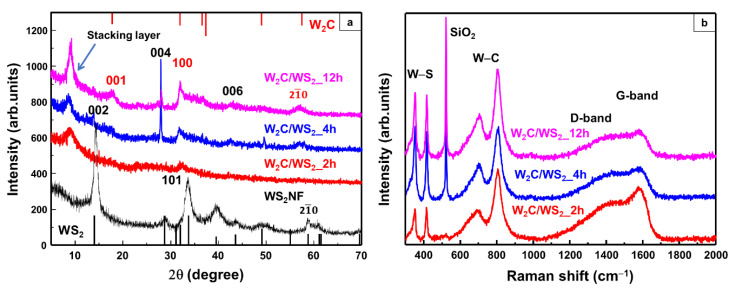
(**a**) X-ray diffraction (XRD) patterns and (**b**) Raman spectra of the W_2_C/WS_2_ alloy nanoflowers (NFs) synthesized by heating at 250 °C for 2, 4, and 12 h.

To further examine the structure of the W_2_C/WS_2_ NFs, powder XRD measurements were carried out over the 2θ range of 5–70° (Figure 2a). The XRD peaks of all the samples could be indexed to the hexagonal structure of W_2_C and WS_2_ [32,33,34,35]. It should be noted that the W_2_C/WS_2__2h sample showed weak WS_2_ peaks. Moreover, this sample showed broad W_2_C peaks, indicating the small crystallite size of W_2_C. The W_2_C/WS_2__4h and W_2_C/WS_2__12h samples showed clear W_2_C and WS_2_ peaks, indicating the co-existence of these hexagonal-structured materials. For comparison, a WS_2_ sample was prepared using the same procedure as that used for preparing the W_2_C/WS_2_ NFs but without the addition of ethanol. This sample showed peaks corresponding to WS_2_ only. Therefore, the addition of ethanol during the synthesis not only prevented the oxidation of WCl_6_ but also contributed to the formation of carbide from the thioacetamide source. The structures of W_2_C and WS_2_ in the alloy NFs were examined by Raman spectroscopy (Figure 2b). The samples for Raman spectroscopy measurements were prepared on SiO_2_/Si substrates, and a 532-nm wavelength laser source was used. The samples showed two peaks at 351.5 and 415.2 cm^−1^ corresponding to the in-plane mode vibration E^1^_2g_ and out-plane mode vibration A_1g_ of WS_2_, respectively [2,36]. Moreover, the peaks detected at 700 and 805 cm^−1^ can be attributed to the stretching modes of W−C [26,37]. The D- and G-bands corresponding to the sp^3^ and sp^2^ carbon atoms in W_2_C were observed at 1200–1700 cm^−1^. These results indicate the co-existence of W_2_C and WS_2_ in the alloy NFs.

**Figure 3:**
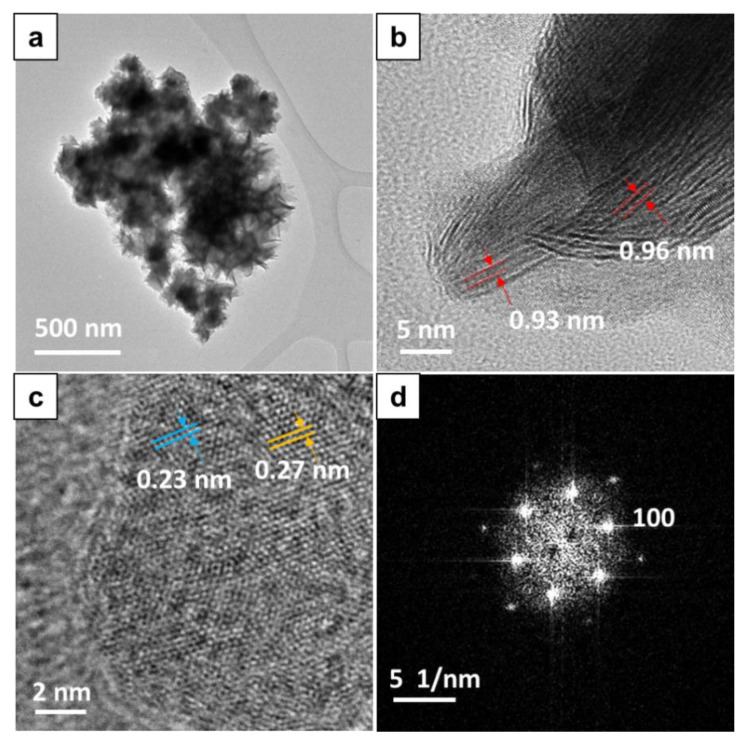
(**a**) TEM image; (**b**,**c**) HRTEM image and (**d**) selected-area electron diffraction (SAED) pattern of the W_2_C/WS_2__4h NF sample.

The structure of the W_2_C/WS_2_ alloy NFs was further examined using TEM. As can be observed from Figure 3a, the samples showed a uniform NF structure with many leaves. Some Moiré patterns with a size of about 0.9–1.0 nm could be observed in the TEM images of the samples (Figure 3b). This can be attributed to the formation of the W_2_C/WS_2_ interlayer spacing [31]. The lattice spacings of W_2_C and WS_2_ in the samples were also measured from their high-resolution TEM (HRTEM) image shown in Figure 3c. The distance spacings of the WS_2_ (100) and W_2_C (101) planes were measured to be 0.27 and 0.23 nm, respectively. These spacings are characteristic of these materials. Moreover, the selected-area electron diffraction (SAED) pattern shown in Figure 3d indicates that W_2_C/WS_2_ alloy NFs with a highly crystalline hexagonal structure were successfully fabricated.

**Figure 4:**
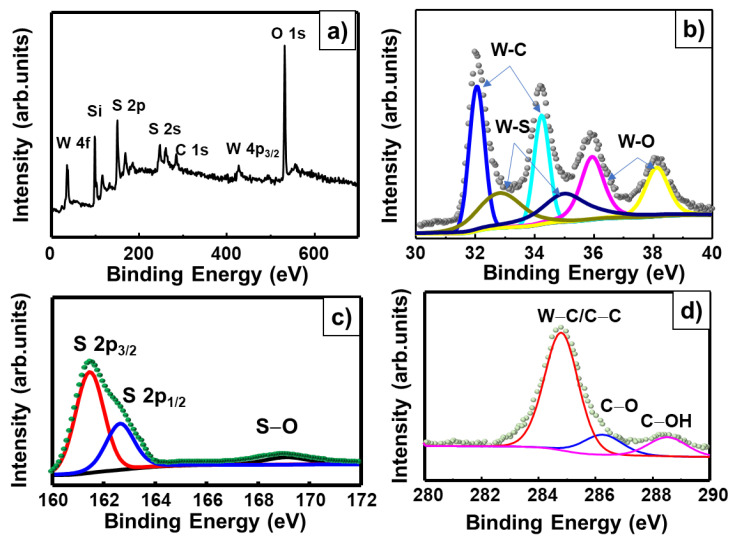
XPS profiles of the W_2_C/WS_2_ _4h NF sample with (**a**) survey scan spectrum and high-resolution (**b**) W 4f, (**c**) S 2p, and (**d**) C 1s spectra.

The chemical compositions and atomic binding energies of the W_2_C/WS_2_ alloys were analyzed by XPS (Figure 4a–d). The survey scan XPS profiles of the alloys showed clear and sharp peaks corresponding to W, S, O, C, and Si (from substrate) (Figure 4a). No impurity peak was detected, indicating the high purity of the W_2_C/WS_2_ alloys. The high-resolution W 4f, S 2p, and C 1s spectra of the alloys are shown in Figure 4b–d, respectively. The W 4f peak consisted of doublet peaks corresponding to the W−C, W−S, and W−O bonds. The contribution of W−O bonding can be attributed to the slight oxidation on the surface of the alloys, which always occurs during the preparation or natural oxidation of a material in air [29]. The W 4f7/2 and W 4f5/2 peaks of the W−C doublet were observed at 31.9 and 34.1 eV, respectively, while those of the W−S doublet were observed at 32.4 and 34.6 eV, respectively. The W 4f7/2 and W 4f5/2 peaks of the W−O doublet were observed at 36.0 and 38.2 eV, respectively. The S 2p peak of the samples could be deconvoluted into the S 2p3/2 and S 2p1/2 peaks, which were observed at 161.4 and 162.6 eV, respectively. Furthermore, the slight oxidation of the surface of the alloys during the preparation resulted in the appearance of the S−O bond peak at about 169 eV. Finally, the C 1s peak of the samples could be deconvoluted into those corresponding to C−W bonding at 284.7 eV, C−O bonding at 286.2 eV, and C−OH bonding at 288.5 eV. This is consistent with the binding energy of C in carbide compounds [38]. 

**Figure 5:**
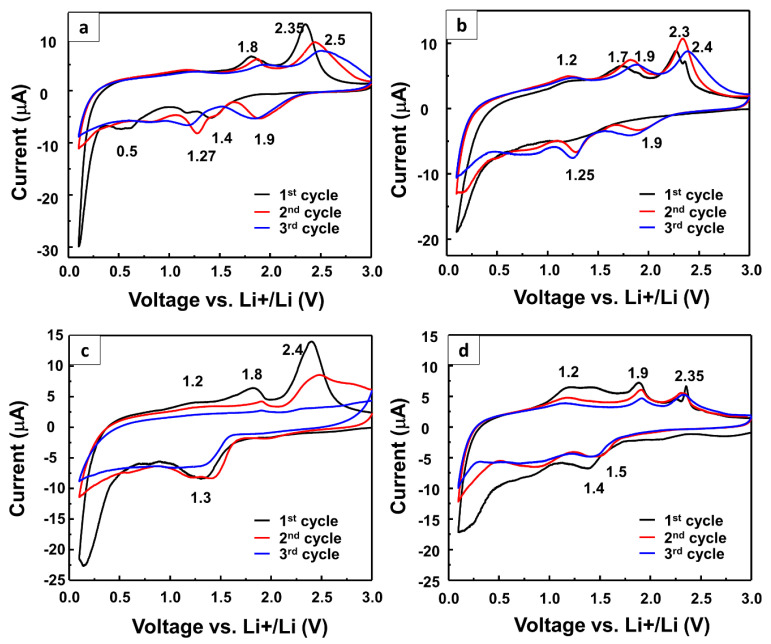
Cyclic voltammograms of the (**a**) as-prepared WS_2_ NF, (**b**)W_2_C/WS_2__2h NF, (**c**)W_2_C/WS_2__4h NF, and (**d**) W_2_C/WS_2__12h NF electrodes.

To evaluate the electrochemical performance of the alloys for application as LIB anode materials, their CV tests were carried out for three cycles over the voltage range of 0.1–3.0 V (Figure 5a–d). In the first cycle, the as-prepared WS_2_NFs showed lithiation at 0.5, 1.2, and 1.4 V attributing to the reduction of WS_2_ to Li_2_S through multiple steps, including the formation of Li_x_WS_2_ and Li_2_S [28]. The peak observed at 0.5 V corresponds to the conversion reaction (4Li^+^ + WS_2_ + 4e^−^ → 2Li_2_S + W) as well as the decomposition of the non-aqueous electrolyte to form the solid electrolyte interface (SEI) layer [28,39]. In the second cycle, the peak at 0.5 V disappeared because of the formation of the SEI and gel-like polymeric layers by the dissolution of Li_2_S into the electrolyte, leading to its degradation [40]. The peaks at 1.2 and 1.4 V shifted to 1.27 V. An additional peak was observed at 1.9 V, indicating the insertion of Li into Li_x_WS_2_ [28]. Figure 5b–d show the cyclic voltammograms of the W_2_C/WS_2__2h, W_2_C/WS_2__4h, and W_2_C/WS_2__12h samples, respectively. In the first cycle, the W_2_C/WS_2__2h sample showed a small peak at about 1.1 V. In the second and third cycles, the sample showed reduction peaks at 1.25 and 1.9 V attributing to the multi-step lithiation of WS_2_. Interestingly, the W_2_C/WS_2__4h and W_2_C/WS_2__12h samples with large NFs showed only a broad peak at 1.3–1.5 V in the first discharge cycle. This phenomenon can be attributed to the change in the work function of WS_2_ by the addition of W_2_C [31]. The changes in the work function of the alloys corresponded to the changes in their reduction potentials. The work function of the W_2_C/WS_2_ NFs increased from 4.31 to 4.7 eV with an increase in the reaction time from 2 to 12 h. The work function of the pure WS_2_ sample was 4.95 eV. The bare WS_2_ electrode showed lithiation peaks at 1.4 and 1.9 V. On the other hand, the W_2_C/WS_2__2h and W_2_C/WS_2__4h electrodes showed lithiation peaks at lower potentials at around 1.2–1.5 V. These samples did not show any lithiation peak at 1.9 V. Hence, it can be stated that the decrease in the work function resulted in a decrease in the lithiation potential of the samples. As shown by the XRD patterns, the W_2_C/WS_2__2h sample showed broader W_2_C peaks than the other samples, indicating that the W_2_C crystallite size of this sample was smaller than those of the other samples. Therefore, the change in the second lithiation potential of the W_2_C/WS_2__2h electrode was comparable to the initial potential of the WS_2_ electrode because of the instability of its smaller W_2_C crystals. The W_2_C/WS_2__4h and W_2_C/WS_2__12h electrodes only showed reduction peaks at about 1.2–1.5 V. This indicates that with an increase in the reaction time, the bonding between W_2_C and WS_2_ became stronger. The W_2_C/WS_2_ and WS_2_ NFs showed similar oxidation peaks because of the restoration of the WS_2_ structure at about 1.7–1.9 V and the oxidation of Li_2_S (Li_2_S → 2Li+S) at about 2.3–2.5 V [39]. The W_2_C/WS_2_ NFs showed an oxidation peak at approximately 1.2 V, which was attributed to the delithiation of W_2_C.

**Figure 6:**
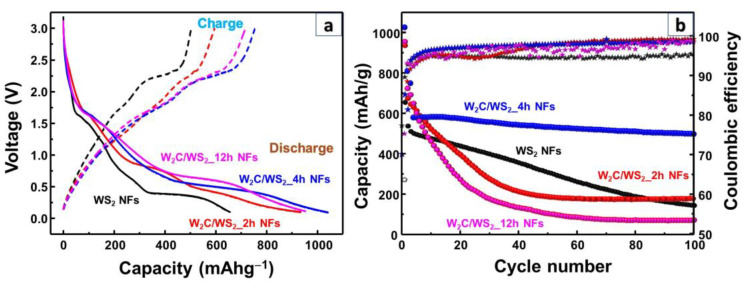
(**a**) Initial voltage profiles and (**b**) cyclic performance of the as-prepared WS_2_ NF, W_2_C/WS_2__2h NF, W_2_C/WS_2__4h NF, and W_2_C/WS_2__12h NF electrodes over 100 cycles.

The initial discharge and charge voltage profiles of the W_2_C/WS_2_ and WS_2_ NFs were obtained over the voltage range of 0.1–3.0 V at a scan rate of 100 mA g^−1^, as shown in Figure 6a. The WS_2_ NFs showed the charge and discharge capacities of 504.0 and 656.6 mAh g^−1^, respectively. The W_2_C/WS_2__2h, W_2_C/WS_2__4h, and W_2_C/WS_2__12h NFs showed the charge and discharge capacities of 595.2 and 935.2 mAh g^−1^, 751.8 and 1040.5 mAh g^−1^, 717.7 and 953.5 mAh g^−1^, respectively. It has been reported that carbide materials are promising candidates for energy storage applications [41]. In this study, the addition of W_2_C to the WS_2_ NF sample improved its storage capacity. The cyclic performances of the W_2_C/WS_2_ and WS_2_ NFs were evaluated over 100 cycles, as shown in Figure 6b. The alloys NF electrodes exhibited different electrochemical properties depending on the reaction time. For instance, after 10 cycles, the capacities of the W_2_C/WS_2__2h and W_2_C/WS_2__12h electrodes decayed rapidly by about 55%. After 30–40 cycles, the samples showed low stable capacities of 100–200 mAh g^−1^ with a high Coulombic efficiency of approximately 95–99%. On the other hand, the W_2_C/WS_2__4h electrode showed excellent cyclic stability with a high capacity of 500 mAh g^−1^ after 100 cycles as compared to the other electrodes. The reasons why the W_2_C/WS_2__4h electrode demonstrated the best performance are the stable binding of W_2_C to WS_2_ and the lowering of reduction potential in addition to the low resistance (discussed later). This sample showed a Coulombic efficiency of 97–98%. Meanwhile, the WS_2_ NFs showed a Coulombic efficiency of 93–94% and a continuous decrease in the cyclic capacity to approximately 145 mAh g^−1^ during the 100th cycle.

**Figure 7:**
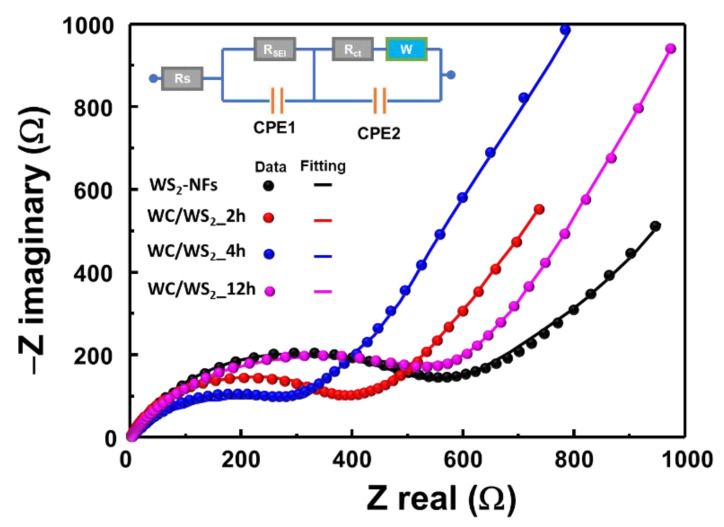
Nyquist plots of the as-prepared WS_2_ NF, W_2_C/WS_2__2h NF, W_2_C/WS_2__4h NF, and W_2_C/WS_2__12h NF electrodes with inset equivalent circuit.

To further investigate the performance of the NFs, their EIS measurements were carried out over the frequency range of 100 kHz–0.1 Hz at a voltage of 3.0 V. Figure 7 shows the Nyquist plots of the WS_2_ and W_2_C/WS_2_ NF electrodes with the equivalent circuit as an inset containing the constant phase elements (CPE1, 2), series resistance (R_s_), charge transfer resistance (R_ct_), solid electrolyte resistance (R_SEI_), and diffusion Warburg element (W). The semicircular arc corresponds to their charge transfer resistances between the interface of electrode and electrolyte [42]. The resistance values are summarized in Table 1. The WS_2_ NF anode showed the highest charge transfer resistance among all the anodes investigated. The charge transfer resistance of the WS_2_ NFs decreased with the addition of W_2_C in the case of the W_2_C/WS_2__2h and W_2_C/WS_2__4h electrodes. However, it increased again for the W_2_C/WS_2__12h electrode. The difference in the performance of the as-prepared W_2_C/WS_2_ electrodes could be due to the different amount of W_2_C in the alloys. As shown by the Raman spectrum in Figure 2b, the peak intensities of the D- and G-band from the carbon atoms in W_2_C decreased when the reaction time increased, illustrating an increase in the amount of WS_2_. In the case of the W_2_C/WS_2__12h sample, it could have an excess amount of H_2_S at 250 °C, and the applied high pressure sulfurized the weak bonding of W−C, leading to the lower amount of C, which was confirmed by Raman spectra representing the decrease of the D-band and G-band. Moreover, the flower size of the W_2_C/WS_2__12h sample is about 1 μm, which is even bigger than the random size of WS_2_ NFs. It should be noted that the increase in size could lead to the decrease in the conductivity [28]. When the amount and size of WS_2_ increase, the resistance of the electrodes increases, as discussed in the EIS results. Specifically, the W_2_C/WS_2__2h and 4h electrodes showed lower resistances compared to that of the W_2_C/WS_2__12h electrode. Even though the W_2_C/WS_2__2h electrode showed the lowest resistance value, the cycling performance was not stable due to the weak binding of the W_2_C crystal to WS_2_, leading to the gradual capacity decay, as discussed earlier. Thus, it can be concluded that the control of reaction time for the preparation of W_2_C/WS_2_ electrodes is crucial to optimize the overall electrochemical properties, where the W_2_C/WS_2_4h alloy is the best electrode. Computational calculations have revealed that W_2_C materials are promising candidates for Li storage applications [30]. Li ions tend to adsorb on W_2_C materials to form a metal cluster. Moreover, MXenes nanosheets materials exhibit high conductivity [43]. In this study, the addition of W_2_C reduced the charge transfer resistance of the WS_2_ NFs, and the resulting W_2_C/WS_2_ alloy NFs showed high storage capacity and stability for LIB applications. 

## 4. Conclusions

W_2_C/WS_2_ NFs were successfully fabricated via a facile hydrothermal method at low temperature. The particle size of the NFs could be controlled (200 nm–1 μm). The obtained NFs exhibited high purity and well-defined hexagonal structures of W_2_C and WS_2_. The NF alloys were employed as anode materials for LIBs. The W_2_C/WS_2__4h sample showed a high initial discharge capacity of 1040 mAh g^−1^. The alloy electrodes showed a low charge transfer resistance of 200–600 Ω, indicating that the W_2_C/WS_2_ alloy electrodes were highly conductive as compared to the WS_2_ NF electrode. Among all the electrodes investigated, the W_2_C/WS_2__4h electrode exhibited the highest stable capacity of up to approximately 500 mAh g^−1^ over 100 cycles, which could be attributed to the optimized W_2_C in WS_2_ NFs, Therefore, the W_2_C/WS_2_ alloy NFs prepared in this study showed a potential for energy storage and conversion applications.

## Figures and Tables

**Table 1:** Comparison of resistance values extracted from equivalent circuit for the electrochemical impedance spectroscopic (EIS) measurement.

Sample	R_s_	R_ct_	R_SEI_
WS_2_ NFs	2.12	627	430
WC/WS_2__2h	3.04	381	367
WC/WS_2__4h	1.24	312	350
WC/WS_2__12h	5.41	620	422
